# Risk factors associated with loss to follow-up from tuberculosis treatment in Tajikistan: a case-control study

**DOI:** 10.1186/s12879-017-2655-7

**Published:** 2017-08-04

**Authors:** Jessica Wohlleben, Mavluda Makhmudova, Firuza Saidova, Shahnoza Azamova, Christina Mergenthaler, Suzanne Verver

**Affiliations:** 1 KNCV Tuberculosis Foundation, Evidence team, The Hague, The Netherlands; 2 Department of International Health, University Maastricht, Maastricht, The Netherlands; 3 KNCV TB Foundation, country office Tajikistan, Dushanbe, Tajikistan; 4 Republican Center for protection of population from TB, Dushanbe, Tajikistan; 5 Present address: Royal Tropical Institute (KIT), Amsterdam, The Netherlands; 6 Academic Medical Centre, AIGHD, Amsterdam, the Netherlands; 7 Present address: Department of Public Health, Erasmus MC, Rotterdam, The Netherlands

**Keywords:** Default, Migration, Case-control study

## Abstract

**Background:**

There are very few studies on reasons for loss to follow-up from TB treatment in Central Asia. This study assessed risk factors for LTFU and compared their occurrence with successfully treated (ST) patients in Tajikistan.

**Methods:**

This study took place in all TB facilities in the 19 districts with at least 5 TB patients registered as loss to follow-up (LTFU) from treatment. With a matched case control design we included all LTFU patients registered in the selected districts in 2011 and 2012 as cases, with ST patients from the same districts being controls. Data were copied from patient records and registers. Conditional logistic regressions were run to analyse associations between collected variables and LTFU as dependent variable.

**Results:**

Three hundred cases were compared to 592 controls. Half of the cases had migrated or moved. In multivariate analysis, risk factors associated with increased LTFU were migration to another country (OR 10.6, 95% CI 6.12–18.4), moving within country (OR 11.0, 95% CI 3.50–34.9), having side effects of treatment (OR 3.67, 95% CI 1.68–8.00) and being previously treated for TB (OR 2.03, 95% CI 1.05–3.93). Medical staff also mentioned patient refusal, stigma and family problems as risk factors.

**Conclusions:**

LTFU of TB patients in Tajikistan is largely a result of migration, and to a lesser extent associated with side-effects and previous treatment. There is a need to strengthen referral between health facilities within Tajikistan and with neighbouring countries and support patients with side effects and/or previous TB to prevent loss to follow-up from treatment.

## Background

TB patients lost to follow-up (LTFU) are defined as “TB patients who did not start treatment or whose treatment was interrupted for two consecutive months or more” [1] and were previously called defaulters. LTFU patients are more likely to redevelop infectious active TB, and are at higher risk of developing MDR-TB [2].

Reasons and risk factors for LTFU have been described extensively, but rarely in countries of the Central Asia Region (CAR) [3–8]. Only two such studies have been reported from Uzbekistan, a quantitative and qualitative one in Tashkent [9, 10]. The quantitative study found that unemployment, being a pensioner, alcoholism and homelessness were associated with default [10]. Few studies on this subject were reported from other former Soviet Union countries, for example from Latvia, [11] Russia [12, 13], Moldova [14], Estonia [15], Armenia [16] and Georgia [17]. They found a large variety of socio-economic and clinical risk factors to be associated with LTFU.

In Tajikistan, no such study has been reported. Although Tajikistan reported a low proportion LTFU among new and retreatment cases in the last few years (4%) [18], this is considered to be an underestimate. Tajikistan reported 77% multidrug-resistant (MDR) TB among previously treated TB cases and 14% MDR TB among newly diagnosed cases in the cohort 2014 [19].This is probably partly a result of incomplete treatment in the past. Therefore it is important to understand the drivers and results of LTFU in Tajikistan to achieve successful treatment outcomes.

This study aimed at determining risk factors for TB patients LTFU and comparing them with patients successfully treated. We also studied factors that have rarely been reported, for example outmigration rather than in-migration, which is a special problem in CAR due to the attraction of labour opportunities in Russia.

## Methods

### Setting

Tajikistan notified to WHO 7035 TB cases in 2011 and 6508 in 2012, of whom 604 and 694 were confirmed MDR cases. The WHO estimated incidence rate in Tajikistan was 87 per 100,000 in 2016. In 2016 a total of 6232 TB patients were registered for treatment in Tajikistan [19].

### Design

We chose a matched case-control design for efficiency, since the proportion of patients LTFU is reported to be only 4%. Patients registered for treatment who became LTFU (as defined by WHO [1]) were defined as cases. Patients diagnosed with TB who did not start treatment are also part of the official WHO definition, but could not be included since they were not registered as such. For each case, two successfully treated (ST) patients following them in the same register were chosen as controls, in effect matching them only on health facility and time of registration (controls had date of registration near that of a case) rather than on the basis of individual characteristics. If any of them was not ST, the next ST patient on the list was chosen as a control.

### Study population

The study population consisted only of TB patients registered for first line treatment in 2011 and 2012, who were marked as defaulters (now called LTFU) or successfully treated in the TB registers. In Tajikistan patients who migrated or moved are defined as LTFU, except when further treatment in a different location is confirmed. Therefore migrated and moved patients marked as LTFU were included as cases in the study. It should be noted that patients who move AND report in another clinic for continuation of treatment, were registered as ‘transfer out’ and have been excluded from this study. About 1% of patients had a treatment outcome registered as ‘transfer out’ in 2011.

### Sampling method and size

Multistage purposeful sampling was used to maximize the number of LTFU patients found visiting a minimum number of facilities. Patients were selected from TB registers located in regional TB centres. Tajikistan has 5 administrative areas: Sughd, Khatlon, region under republican sub-ordination, Dushanbe and Gorno-Badakhshan Autonomous region (GBAO), with together 59 districts. We selected all districts that each had at least five LTFU DS-TB patients registered for treatment in 2011 [18]. These were 19 districts within 4 administrative areas (only 1 patient LTFU was registered in GBAO in 2011 and this whole region was excluded).

### Data collection and validation

Data collection took place in all 34 TB centres between May and June 2014 in the 19 selected districts. Information was taken from TB registers and individual patient records, while facility medical staff were consulted when additional information was needed or when information in registers contradicted patient records.^1^ The risk factors measured in the study were those registered in the patient records. Factors such as homelessness and alcoholism had no systematic definition in the patient records, and were based on the observations made by TB clinicians.

An electronic data collection form was developed using the Russian translation of EpiData [20] to obtain information from the TB registers TB03/TB0 3 U, TB01/TB0 1 U. [http://www.who.int/tb/dots/r_and_r_forms/en/], patient records and laboratory registers from TB centres. The tool enabled the direct entry of data extracted from paper documents in an electronic database. To ensure quality, the data entry form was tested in a pilot by two of the researchers, who also participated during the actual data collection to enter data.

Data have been collected by trained study clinicians and epidemiologists in 4 teams of 2 persons each. During the course of data collection the teams held contact via phone in order to solve problems concerning the use of different codes and other queries, ensuring consistency between the teams.

We also asked medical staff (the treating clinician and nurse, whoever knew most about the patient) what was the reason for LTFU according to the health care provider, for each individual patient LTFU.

### Data analysis

For data cleaning and analysis the database was exported from EpiData into SPSS 21 for Windows (SPSS, Inc., Chicago, USA). First univariate conditional logistic regressions were run to analyse associations between collected variables and LTFU as dependent variable. Since each case had more than one control the Cox regression command was used to perform conditional logistic regression. Variables with significant associations in the univariate models were included in a multivariate conditional logistic regression model using the likelihood ratio with backward selection of independent variables. 95% confidence intervals (CI) were calculated for all variables included in the univariate and multivariate regressions.

### Permission and informed consent

Official permission to conduct this study was issued by the National Ethics Committee of the Ministry of Health (MoH) Tajikistan, and all facility directors gave permission to participate prior to data collection. Data from TB registers, patient medical records and laboratory registers could only be matched by patient names, which were deleted immediately after linking was completed.

## Results

### Selection and characteristics of study population

According to the national TB database, 503 patients were LTFU in Tajikistan among the cohorts diagnosed and on first line treatment in 2011 and 2012 [20] (Table 1). This is 4% of the cases notified to WHO whom did not have confirmed MDR (6431 and 5814, respectively). Among them 334 TB patients (64%) were registered in the 19 districts. Two controls were selected per case, therefore 644 ST patients were expected. Only 300 (89.8%) of the expected 334 LTFU patients were actually found in the registers. The others had successful or unclear treatment outcomes or errors in registers such as a missing final sputum test which was not actually missing but just not entered in the register. Only 83.9% of reported LTFU patients could be retrieved from the registers in 2011 and 95.4% in 2012. The 300 LTFU cases have been matched with 644 successfully treated patients (controls) of whom 592 had complete enough data to include them (91.9%).

**Table 1:** Selection of patients and those actually found

	Cases (LTFU)			2 controls (ST) per case
	2011	2012	Total	Total
Registered in national database	DS TB	DS TB		
Country	241	262	503	
19 Selected Districts with > = 5 patients LTFU	161 (66.8%)	173 (66.0%)	334 (66.4%)	644
Found in registers	135 (83.9%)	165 (95.4%)	300 (89.8%)	592 (91.9%)

The majority of ST and LTFU patients in the study population were male (*n* = 316; 53.4%, *n* = 202; 67.3% respectively), between 19 and 40 years of age (*n* = 316; 53.4%, *n* = 202; 67.3%), living in rural areas (*n* = 455; 76.9%, *n* = 236; 78.7%), had PTB (*n* = 434; 73.3%, *n* = 253; 84.3%), of which slightly less than half were confirmed smear-positive at baseline (*n* = 229; 38.7%, *n* = 140; 46.7%) (Table 2). Only 9.2% of all TB patients registered in 2011 were tested for drug resistance at time of diagnosis.

**Table 2:** Univariate analyses of factors associated with loss to follow-up

Factors	Loss to follow-up		Successful Treated		Crude Odds Ratio	CI (95%)
	N	Column %	N	Column %		
Totals	300		592			
Sex						
Males	202	67.3	316	53.4	1.78	1.33–2.40
Females	98	32.7	276	46.6	ref	
Age group						
0–18	19	6.3	83	14.0	0.36	0.21–0.62
19–40	202	67.3	316	53.4	ref	
41–60	57	19.0	136	23.0	0.68	0.47–0.97
>60	21	7.0	57	9.6	0.55	0.32–0.95
Unknown	1	0.3				
Place of Living						
Rural	236	78.7	455	76.9	ref	
Urban	56	18.7	132	22.3	0.59	0.29–1.20
Unknown	8	2.7	5	0.8		
Baseline smear result						
Baseline smear negative	112	37.2	212	35.8	ref	
Baseline smear positive	140	46.7	229	38.7	1.15	0.83–1.59
Baseline smear not done	48	16.1	150	25.3	0.57	0.37–0.86
Unknown			1	0.2		
Location of TB						
PTB	253	84.3	434	73.3	ref	
EPTB	47	15.7	158	26.7	0.49	0.34–0.72
Patient category						
New	211	70.3	495	83.6	Ref	
Previously treated	40	13.3	34	5.7	2.86	1.70–4.82
Transferred in	2	0.7	4	0.7	1.39	0.20–9.48
Other	47	15.7	58	9.8	1.96	1.27–3.02
Unknown			1	0.2		
Pregnancy						
Pregnancy no	75	25.0	202	34.1	ref	
Pregnancy yes	3	1.0	2	0.3	65.3	0 - >100
Pregnancy na.	217	72.3	371	62.7		
Unknown	5	1.7	17	2.5		
Side effects						
Side effects no	237	79.0	520	87.8	ref	
Side effects yes	26	8.7	19	3.2	3.22	1.64–6.33
Unknown	37	12.3	53	9.0		
Moved within country						
Moved no	264	88.0	570	96.3	ref	
Moved yes	26	8.7	7	1.2	10.7	4.08–28.1
Unknown	10	3.3	15	2.5		
Seasonal work						
Seasonal work no	283	94.3	568	95.9	ref	
Seasonal work yes	6	2.0	11	1.9	1.02	0.36–2.90
Unknown	11	3,7	13	2.1		
Migration other country						
Migration no	167	55.7	534	90.2	ref	
Migration yes	123	41.0	44	7.4	9.70	6.06–15.5
Unknown	10	3.3	14	2.4		
Imprisonment ever						
Imprisonment no/unknown	292	96.8	585	98.8	ref	
Imprisonment yes	8	3.2	7	1.2	2.19	0.74–6.46
Alcoholism						
Alcoholism no	247	82.3	540	91.2	ref	
Alcoholism yes	13	4.4	14	2.4	1.87	0.82–4.28
Unknown	40	13.3	38	6.4		
Drug abuse						
Drug abuse no	253	84.3	551	93.1	ref	
Drug abuse yes	6	2.0	3	0.5	5.21	1.02–26.6
Unknown	41	13.7	38	6.4		
Employment						
Unemployed	237	79.0	364	61.5	ref	
Working	17	5.7	39	6.6	0.68	0.36–1.27
Student	6	2	23	3.9	0.40	0.16–1.05
Pensioner	18	6.0	55	9.3	0.51	0.27–0.93
Not applicable	17	5.7	73	12.3		
Unknown	5	1.7	38	6.4		
Education						
Middle school	249	83.0	432	73.0	ref	
College	4	1.3	19	3.2	0.51	0.16–1.58
University	8	2.7	16	2.7	0.84	0.35–2.04
Still studying	11	3.7	45	7.6	0.45	0.23–0.89
No degree	11	3.7	30	5.1	0.57	0.26–1.24
Unknown	17	5.6	50	8.4		
Homelessness						
Homelessness no	281	93.7	575	97.1	ref	
Homelessness yes	15	5.0	15	2.5	2.63	1.19–5.82
Unknown	4	1.3	2	0.3		
Hospitalization						
ambulatory	146	48.7	292	49.3	ref	
hospital	152	50.7	296	50.0	1.07	0.78–1.47
Different country	1	0.3	3	0.5	0.75	0.08–7.23
Prison hospital	1	0.3	1	0.2	2.58	0.16–42.3

Ref = reference category

The majority (69%) of ST patients were smear-negative at end of treatment, the others had unknown smear status. Among those LTFU, the proportion of those who were lost to follow up from TB treatment during the intensive treatment phase (34%) was lower than during the continuation phase (59%), the remainder was unknown.

### Factors associated with loss to follow-up

#### Univariate analysis

The univariate analysis showed that being male (OR = 1.78; 95% CI 1.33–2.40), having side effects (OR = 3.22; CI = 1.64–6.33), being previously treated for TB (OR = 2.86; CI = 1.70–4.82), patient category other (OR = 1.96; CI = 1.27–3.02), having moved within country (OR = 10.7; CI = 4.08–28.1) or migrating out of the country (OR = 9.70; CI = 6.06–15.5), homelessness (OR 2.63, CI = 1.19–5.82) and ever abusing drugs (OR = 5.21; CI = 1.02–26.6) were significantly positively associated with LTFU from treatment (Table 2). The strongest risk factor was moving within or out of country, both as compared to not moving. Forty percent of patients LTFU migrated out of country and 9% within country.

All age groups had a significantly lower risk of LTFU when compared to the largest age group of 19–40 years (Table 2). Other groups who have a lower risk of LTFU were those whose baseline smear was not performed (OR = 0.57; CI = 0.37–0.86), those having EPTB (OR = 0.49; CI = 0.34–0.72), those still being in school (OR = 0.45; CI = 0.23–0.89) and pensioners (OR = 0.51; CI = 0.27–0.93).

Other factors not significantly associated with LTFU were rural/urban place of living, seasonal work, alcohol abuse, employment, pregnancy, homelessness and hospitalization during intensive treatment phase (Table 2).

For type of drug-resistance an HIV there were too few patients with test results to do a meaningful analysis.

#### Multivariate analysis

The factors sex, age group, TB location, side effects, patient category, migration out of Tajikistan, and moved within country were included in the stepwise model for the multivariate analysis. Out of these factors, only having side effects (OR = 3.67; CI = 1.68–8.00), being previously treated (OR = 2.3; CI = 1.05–3.93), moved within country (OR = 11.04; CI = 3.50–34.9) and migration to another country (OR = 10.6; CI = 6.12–18.4) had a significant association with LTFU (Table 3). As in the univariate analyses the association was strongest for the factors moved within country and migration to another country, and with slightly higher odds ratios.

**Table 3:** Factors associated with loss to follow-up in multivariate analysis

Factor	N^a^	Adjusted Odds Ratio	CI (95%)
Side effects			
Side effects no	769	ref	
Side Effects yes	69	3.67	1.68–8.00
Patient category			
New case	646	ref	
Retreatment case	96	2.03	1.05–3.93
Other	94	1.65	0.89–3.06
Moved within country			
Moved no	808	ref	
Moved yes	28	11.04	3.50–34.9
Migration other country			
Migration no	676	Ref	
Migration yes	160	10.6	6.12–18.4

^a^ *n* = 836, due to exclusion of those with missing values

Medical staff mentioned a reason for LTFU for 292 out of 317 (92%) patients LTFU. They confirmed that the majority of TB patients was lost to follow-up due to migration to another country (50% of 292) or within Tajikistan (10%), with other reasons mentioned often refusing further treatment (15%), and side effects (8%), stigma (5%) and family problems (5%).

## Discussion

We found that the main factors associated with LTFU were migration out of country, moving within country, side effects and previous treatment. Medical staff confirmed these but also mentioned patients refusing further treatment, stigma and family problems.

### Treatment phase during LTFU

LTFU occurred more often in the continution phase (58.8%) than in the intensive phase of treatment (34.2%). Conversely, an older study (2007) on default in Uzbekistan found that the majority of LTFU cases interrupted treatment in the intensive phase (62%) [10]. At the time of our study, Tajikistan was more advanced in decentralizing services to primary health care facilities than was Uzbekistan; therefore, patients in Tajikistan may have stayed on treatment longer.

### Risk factors

We found the main risk factor for LTFU in Tajikistan was migration to another country. To our knowledge, this has not been reported before, except in Moldova, where spending more than three months abroad within the past year was a risk factor for LTFU. For example a similar study in Uzbekistan did not find this [10]. Many Tajiks migrate to Russia for work. Often when during migration they are diagnosed with TB, they are sent home for treatment. TB treatment in Tajikistan means a substantial drop in financial support for families due to lost wages. Although TB treatment and diagnosis should be free of charge, certain diagnostic procedures and additional medication accompanying TB treatment often have to be paid out of pocket. This is a major burden for patients and their families and increases the motivation to leave the country for work again even if patients have not successfully completed treatment [21].

In contrast to the situation in Central Asia, studies globally found a relationship between being an immigrant coming from another country and LTFU [13, 23–28]. Most of those studies were conducted in European Union (EU) member states or USA where over a third of the reported TB cases are immigrants. In combination with our study, this shows that migration - regardless whether out of or into a country or within a country- may inhibit completion of TB treatment.

For the variable patient category, being previously treated showed a significant positive association with LTFU, as was found elsewhere [14, 15, 17, 26]. This means that patients who already received TB treatment were more likely to be LTFU from subsequent treatments, including patients with a relapse, former treatment failure, after LTFU, and transferred patients.

Our finding that side effects were associated with loss to follow-up, was also found by others [15]. Most studies do not report on an association between LTFU and side effects, possibly since side-effects are often not recorded in a standard way in TB records [4]. An association with LTFU is expected for individuals taking MDR-TB drugs since second line drugs are more toxic and more likely to cause severe side effects.

Many studies have found other factors significantly associated with LTFU, such as alcohol use and drug addiction [12, 14, 16, 22, 29–31]. We could not confirm these, possibly since the factor migration was so strong in our study population. Another reason may be that these factors may not have been noted accurately since alcohol and drug abuse are not socially acceptable in the local culture. Even though every patient diagnosed with TB is tested for HIV as of 2010, many physicians do not note this in the TB registers [32].

Another risk factor that was found to be significant in many other studies but not significant at all in ours was hospitalization versus ambulatory treatment [10, 30, 33]. Hospitalization during intensive phase is common practice in Tajikistan. Possibly the result of the analysis is related to a clear pre-selection of patients to be hospitalized in Tajikistan since 2011–2012. Alternatively, we may not have measured the actual risk factor, which is: being released from hospitalization to ambulatory care without proper follow-up [10, 30].

### Reasons for LTFU given by medical staff

The factors mentioned by the clinical staff, such as stigma, family problems and patient refusing to take more treatment, were within FSU countries also found in Uzbekistan and Armenia [9, 10, 15]. We also attempted in-depth interviews in a later cohort that was LTFU and could interview only 20 of the patients loss to follow-up. Half of those patients still had TB and 6/20 thought they had completed treatment. Although these numbers low, they indicate that there may be communication issues between medical staff and patients on treatment completion.

### Strength and limitations

The strength of this study was that it was the first in its kind reported from Tajikistan, and the only recent one from the central Asian region, included a large number of patients and is largely generalizable to the country. A limitation was that only variables available in medical records could be analyzed. Further drug sensitivity testing was not standard yet at time of the study. Lastly, it is possible that answers from medical staff on reasons for LTFU were influenced by recall bias.

## Conclusions

In conclusion, we found migration to another country, moving within country, side effects of treatment and previous treatment to be significant risk factors for LTFU. In order to improve follow-up for patients moving within the country it is important to increase the communication and exchange of referral documents between health facilities. A recent meta-analysis showed that psycho-emotional support and socio-economic support were associated with a significant improvement of successful treatment outcomes [34]. Such support may motivate patients to postpone their migration until after treatment completion. Community health workers may assist to reduce LTFU [23]. Expanding international measures such as WHO Europe’s ‘minimum package for cross-border TB control and care’ is crucial in order to improve the situation of TB patients migrating to other countries or those falling sick abroad [35]. Careful monitoring and management of side effects is needed and patients who have been previously treated need extra care to ensure treatment completion.

## Abbreviations

DS TB: Drug Sensitive Tuberculosis
EPTB: Extra Pulmonary Tuberculosis
HCW: Health Care Worker
LTFU: Loss to follow up
MDR TB: Multi-Drug-Resistant Tuberculosis
MoH: Ministry of Health
PTB: Pulmonary Tuberculosis
ST: Successful Treatment
TB 01 U: Multidrug-Resistant Tuberculosis patient cards
TB 01: Drug Sensitive Tuberculosis patient cards
TB 03 U: Multidrug-Resistant Tuberculosis register
TB 03: Drug Sensitive Tuberculosis register
TB: Tuberculosis
XDR TB: Extensively-Drug-Resistant Tuberculosis

## Footnotes

1 The definitions for TB cases and treatment outcomes used in this paper are according to the revised WHO definitions^1^. However, the study population consists of patients registered in 2011 and 2012 when the new definitions were not yet implemented.
